# Enhanced ASR Mitigation and Carbon Reduction Potential of Local Natural Pozzolans as Alternatives to Fly Ash in Cement-Based Systems

**DOI:** 10.3390/ma19143043

**Published:** 2026-07-15

**Authors:** Li Yang, Ming Ma, Zuquan Jin, Fengyin Du

**Affiliations:** 1 School of Civil and Safety Engineering, Wanjiang University of Technology, Ma’anshan 243011, China; 2 Engineering Research Center of Concrete Technology Under Marine Environment, Ministry of Education, Qingdao 266033, China; 3 College of Civil Engineering, Qingdao University of Technology, Qingdao 266033, China; 4 School of Materials Science and Engineering, Southeast University, Nanjing 211189, China

**Keywords:** natural pozzolans, alkali–silica reaction, supplementary cementitious materials, fly ash replacement, life cycle assessment, low-carbon cement

## Abstract

**Highlights:**

- Local natural pozzolans outperformed Class F fly ash in Alkali-Silica Reaction (ASR) mitigation.
- Natural pozzolans (NP) 1 and NP3 maintained ASR expansion below the 0.10% threshold.
- NPs significantly reduced ASR-induced surface cracking.
- NP systems achieved up to 83% of Original Portland cement compressive strength at 91 days.
- Up to ~20% embodied carbon reduction was achieved through clinker replacement.

**Abstract:**

The declining availability of fly ash has intensified the need to identify alternative supplementary cementitious materials (SCMs) capable of maintaining engineering performance while improving durability and reducing greenhouse gas (GHG) emissions. This study evaluates three locally sourced natural pozzolans (NPs) as potential regional alternatives to fly ash (FA), with particular emphasis on alkali–silica reaction (ASR) mitigation performance, hydration behavior, mechanical properties, setting characteristics, and embodied carbon reduction. Physical and chemical characterization revealed high silica contents (65–71%) and relatively fine particle size distributions (d50 between 10 and 14 μm). Accelerated mortar bar testing demonstrated that the natural pozzolans provided substantially greater ASR mitigation than FA. While the FA mixtures continued to exhibit noticeable expansion growth during the testing period, NP1 and NP3 maintained expansion values near or below the commonly used 0.10% mitigation threshold and exhibited significantly reduced visible surface cracking, indicating superior resistance to ASR-related deterioration. Isothermal calorimetry indicated slightly lower early-age heat release for the NP systems compared with OPC and FA, reflecting reduced clinker content and moderate pozzolanic reactivity. Although the natural pozzolans generally exhibited lower early-age strength and stiffness than FA, all NP systems demonstrated continuous long-term mechanical development. At 91 days, compressive strength and dynamic modulus reached up to 83% and 92% of OPC, respectively, with NP1 showing the closest overall mechanical performance to FA. In contrast to FA, the natural pozzolans accelerated both initial and final setting times. A cradle-to-gate life cycle assessment further showed that SCM incorporation significantly reduced embodied carbon emissions, with natural pozzolans achieving greater carbon reduction than FA at equivalent replacement levels. Overall, the results demonstrate that locally available natural pozzolans, particularly NP1 and NP3, can serve as promising alternatives to fly ash by combining superior ASR mitigation performance, meaningful long-term mechanical properties, and substantial carbon reduction potential.

## 1. Introduction

Climate warming has been exacerbated at an unprecedented rate over the past two millennia. In terms of carbon dioxide emissions, the increase in atmospheric CO_2_ concentration between 1900 and 2019 represents a growth rate approximately four times higher than the average rate observed over the past 56 million years [1,2,3,4]. To mitigate the potentially catastrophic impacts of climate change on both human society and natural ecosystems, the Paris Agreement established the goal of limiting global average temperature rise to well below 2 °C above pre-industrial levels, while pursuing efforts to restrict warming to 1.5 °C. Achieving this target requires a rapid and substantial reduction in greenhouse gas (GHG) emissions. According to carbon budget analyses, global emissions must decline by approximately 45% from 2010 levels by 2030 and reach net-zero around mid-century [5,6,7]. China has committed to reaching peak CO_2_ emissions before 2030 and achieving carbon neutrality by 2060. Meeting these ambitious goals demands profound transformation of both the energy structure and industrial systems, particularly in high-emission sectors such as cement production [6,8,9,10,11].

Climate change has intensified the need to reduce greenhouse gas (GHG) emissions from high-carbon industries, among which cement production is one of the largest contributors due to the energy-intensive clinker manufacturing process and limestone calcination [1,2,3,4]. Partial replacement of clinker with supplementary cementitious materials (SCMs) is widely recognized as one of the most effective strategies for reducing the embodied carbon of cement-based materials while maintaining engineering performance [12,13,14,15,16,17]. Among commercially available SCMs, Class F fly ash has been extensively used because of its favorable pozzolanic activity, durability enhancement, and contribution to long-term strength development. However, the global transition away from coal-fired power generation has led to a rapid decline in fly ash availability, creating an urgent need for alternative SCMs that are locally available, technically effective, and environmentally sustainable [18,19,20,21,22,23,24,25,26].

Natural pozzolans have attracted increasing attention as potential fly ash alternatives because they are abundant in many regions and can substantially reduce clinker consumption and associated carbon emissions. Nevertheless, unlike fly ash, the performance of natural pozzolans varies considerably depending on mineralogy, amorphous phase content, fineness, and geological origin [18,27,28,29,30,31]. Studies from different regions have demonstrated that volcanic tuffs from Turkey, volcanic ash from the 2021 La Palma eruption in Spain, and basaltic natural pozzolans from Saudi Arabia all satisfy the basic requirements for cementitious applications while exhibiting different levels of pozzolanic activity, strength development, and durability improvement [32,33,34]. Çavdar and Yetgin [32] investigated six types of volcanic tuff specimens collected from the Trabzon and Bayburt regions of northeastern Turkey and found that all samples met the pozzolanic activity requirements of TS 25, with 7-day compressive strengths ranging from 6.7 to 11.0 MPa. Their results further demonstrated a strong positive correlation between SiO_2_ content and pozzolanic activity, suggesting that silica-rich tuffs are particularly suitable for use as natural pozzolans in cement systems. In a different volcanic context, Sanjuán et al. [33] evaluated ash generated by the 2021 Cumbre Vieja eruption on La Palma (Canary Islands, Spain) as a Portland cement constituent. The La Palma ash exhibited a sum of acid oxides (SiO_2_ + Al_2_O_3_ + Fe_2_O_3_) exceeding 70%, meeting ASTM C618 requirements [35], and blended cements with up to 40% replacement content satisfied standardized chemical, physical, and mechanical requirements while showing improved resistivity and reduced capillary absorption at later ages. The strength activity index at 28 days reached 84.3%, meeting EN 450-1 criteria [36], although compressive strength decreased progressively with increasing replacement level. In the context of arid regions, Khan and Alhozaimy [34] investigated locally available natural pozzolans from basalt plateaus in Saudi Arabia, sourced from four different locations. Despite differences in source, the chemical and physical properties of the Saudi natural pozzolan were consistent across sources and conformed to ASTM C618 Class N requirements, with SiO_2_ + Al_2_O_3_ + Fe_2_O_3_ contents ranging from 73.2% to 74.8%. Concrete containing up to 15% natural pozzolan demonstrated strength activity indices of 77% and 78% at 7 and 28 days, respectively, and the mix was found to approach the performance of concrete containing 20% Class F fly ash. Furthermore, the chloride permeability of natural pozzolan concrete decreased significantly at ages beyond 90 days, reflecting continued pozzolanic reaction and pore structure refinement. These studies collectively demonstrate that locally available natural pozzolans—whether volcanic tuffs, eruption ash, or basaltic scoria—can serve as viable and sustainable SCMs, though their suitability is strongly governed by source mineralogy, SiO_2_ content, fineness, and replacement level. Critically, the majority of existing studies have focused on a limited subset of performance metrics, and direct comparisons between locally sourced natural pozzolans and Class F fly ash encompassing alkali–silica reaction (ASR) mitigation, hydration behavior, mechanical properties, setting characteristics, and embodied carbon within a unified experimental framework remain scarce. These findings confirm the potential of locally sourced natural pozzolans as sustainable SCMs, but also highlight that their performance cannot be generalized across different geological sources.

Several studies have directly compared natural pozzolans with Class F fly ash. Sharbaf et al. [37] reported comparable long-term mechanical and transport performance of natural pozzolan and fly ash in self-consolidating concrete, while Mohsen et al. [38] and Abu-Jdayil et al. [39] investigated the durability and mechanical behavior of natural zeolite-containing systems. More recently, Mousavinezhad et al. [40] evaluated ASR mitigation together with mechanical and durability performance of a locally sourced pumicite. Although these studies demonstrated that natural pozzolans can perform similarly to fly ash under certain conditions, each focused on only a subset of material properties. Consequently, direct comparisons that simultaneously consider hydration behavior, setting characteristics, ASR mitigation, mechanical performance, and environmental impacts within a unified experimental framework remain limited.

Among the durability benefits provided by SCMs, mitigation of alkali–silica reaction (ASR) is particularly important for transportation infrastructure containing reactive aggregates. Previous studies have shown that fly ash and other SCMs reduce ASR expansion primarily through alkali dilution, calcium hydroxide consumption, pore solution modification, and microstructural refinement [41,42,43,44,45,46,47]. Natural pozzolans have also demonstrated promising ASR mitigation; however, their effectiveness depends strongly on mineralogical composition, reactive silica content, particle fineness, and replacement level. Furthermore, although pozzolanic reactivity is commonly used to evaluate SCM performance, the relationship between reactivity and ASR mitigation has not been systematically established for locally sourced natural pozzolans.

To address these knowledge gaps, this study systematically evaluates three locally sourced natural pozzolans from eastern China (tephra ash, zeolite, and pumice) as direct alternatives to Class F fly ash. A unified experimental framework was developed to compare physical and chemical characteristics, pozzolanic reactivity, hydration kinetics, setting behavior, ASR mitigation, compressive strength, dynamic modulus, and cradle-to-gate embodied carbon. Beyond providing a comprehensive comparison, this study demonstrates that locally sourced natural pozzolans can provide substantially greater ASR mitigation than Class F fly ash despite exhibiting lower overall pozzolanic reactivity. This finding indicates that chemical composition and particle characteristics can be more influential than DOR* alone in governing ASR resistance. The results provide both mechanistic insight into SCM performance and practical guidance for selecting locally available natural pozzolans as sustainable fly ash alternatives for transportation infrastructure.

## 2. Materials and Methods

### 2.1. Materials

**Figure 1:**
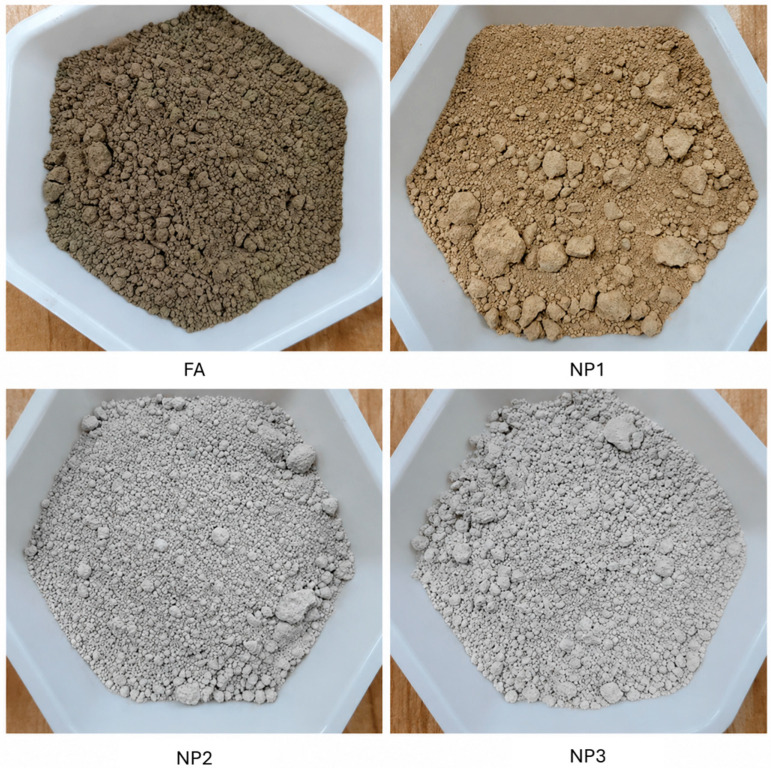
The photos of SCMs.

The cement used in this study was P. I ordinary Portland cement (OPC) commercially available from Sinopharm Chemical Reagent Co., Ltd. (Nanjing, China). Standard class F fly ash (FA) was used in this study to compare with natural pozzolans. Three natural pozzolans are locally sourced from Nanjing, China and they are tephra ash (NP1), zeplite (NP2) and pumice (NP3), respectively, as shown in Figure 1. Following sealing and 24 h curing, all demolded cement pastes were subjected to standard curing conditions (i.e., relative humidity ≥ 95% and ambient temperature of 20 ± 2 °C).

### 2.2. Mixing Procedure

The paste mixture proportions are summarized in Table 1. All mixtures were prepared with a constant water-to-binder ratio of 0.45. Because the investigated supplementary cementitious materials have lower specific gravities than ordinary Portland cement (as confirmed in Section 3.1), replacing cement on an equal mass basis would increase the binder volume and alter the volumetric proportions of the paste. Such changes would influence particle packing, the water-to-solid volume ratio, and pore structure, making it difficult to isolate the intrinsic effects of the SCMs. Therefore, all mixtures were proportioned using an equivalent-volume replacement of OPC. This approach maintains a constant binder volume across all mixtures, allowing differences in hydration behavior, setting characteristics, mechanical properties, and durability to be attributed primarily to the intrinsic properties of the SCMs rather than changes in mixture volumetrics.

### 2.3. Methodology

The specific gravity of the SCMs was measured using a pycnometer procedure. Each SCM sample was tested in three replicates. The loss on ignition (LOI) of the cement, fly ash-F, and natural pozzolans was determined in accordance with GB/T 176-2025 [48]. The chemical compositions of the cement and SCMs were characterized using X-ray fluorescence (XRF) spectroscopy. Three replicate measurements were performed for each sample. Particle size distributions of OPC and SCMs were measured using a Horiba LA-960 laser diffraction analyzer. The pozzolanic reactivity of the SCMs was evaluated using the DOR* approach described in previous studies [49,50,51,52]. DOR* represents the maximum fraction of SCM capable of reacting with calcium hydroxide under the selected testing conditions. The method combines calcium hydroxide consumption determined by thermogravimetric analysis (TGA) with cumulative heat release measured by isothermal calorimetry. SCM and reagent-grade CH were dry blended at a mass ratio of 1:3 and mixed with 0.5 M KOH solution [49,53]. Approximately 7 g of paste was sealed in glass ampoules and stored in a TAM Air calorimeter at 50 ± 2 °C for 240 h. After calorimetry, samples were analyzed using TGA to quantify CH consumption [52,54,55]. Hydration behavior was characterized using isothermal conduction calorimetry (TAM Air, TA Instruments, New Castle, DE, USA). Paste mixtures with a w/b ratio of 0.45 were prepared and approximately 9 g of material was sealed in 20 mL ampoules. The samples were externally mixed for 2 min and transferred immediately to the calorimeter.

The calorimeter was maintained at 23 ± 0.1 °C, and heat evolution was monitored continuously for 7 days. Quartz was used as the reference material. Three replicate measurements were performed for each mixture. Initial and final setting times were determined in accordance with GB/T 1346-2011 [56]. Paste mixtures containing OPC or OPC with 20 vol% SCM replacement were prepared at a constant w/b ratio of 0.45.

Initial setting time was defined as the elapsed time corresponding to a penetration depth of 25 mm, while final setting time was determined when the penetration depth became less than 1 mm. The alkali–silica reaction (ASR) mitigation performance of the SCMs was evaluated using the Accelerated Mortar Bar Test (AMBT) in accordance with ASTM C1567 [41]. Most SCMs were investigated at two replacement levels, while several materials were tested at three levels to determine the dosage required for ASR mitigation.

Initially, all SCMs were evaluated at 32% volumetric replacement. Additional replacement levels were selected based on the measured expansion behavior. Bishop reactive fine aggregate (R3 classification according to ASTM C1778 [57]) was used in this study [58]. The aggregate mainly consisted of granitic rock, quartz, feldspar, diorite, and volcanic materials containing strained quartz and volcanic glass susceptible to ASR.

Mortar bars with dimensions of 25.4 × 25.4 × 285 mm were prepared following ASTM C1567 and stored in 1 N NaOH solution at 80 °C. Expansion measurements were monitored up to 28 days using a length comparator. Three specimens were tested for each mixture, and the average expansion was reported.

According to ASTM C1567, mixtures exhibiting 14-day expansions below 0.10% are considered effective in mitigating deleterious ASR expansion.

**Figure 2:**
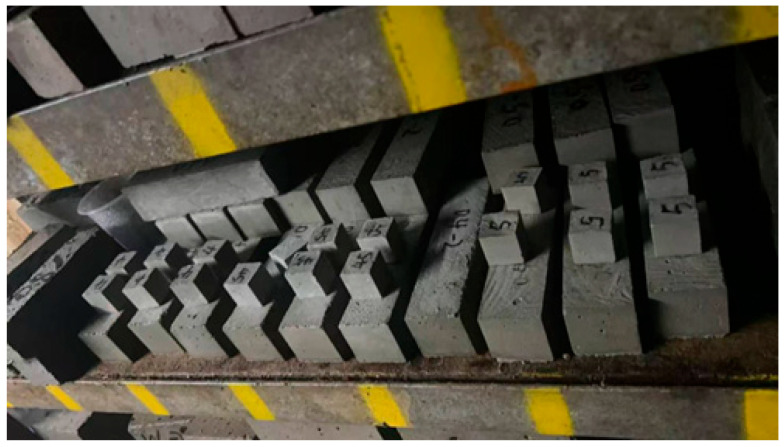
The photos of curing samples.

**Figure 3:**
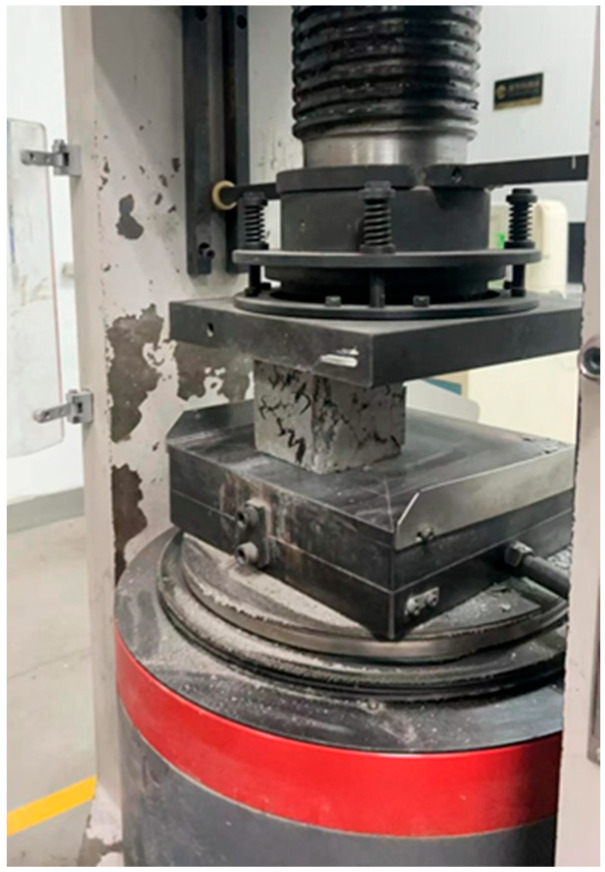
The standard experiment set up for compressive strength.

Compressive strength tests were conducted on mortar specimens prepared according to GB/T 50107-2010 [59]. The mixture proportions are shown in Table 2. The specimens were cured at 23 ± 1 °C as shown in Figure 2. Figure 3 presents the standard compressive strength testing procedure. After demolding, compressive strength measurements were performed at 1, 3, 7, 28, 91 days following the procedures specified in the standard [60].

**Figure 4:**
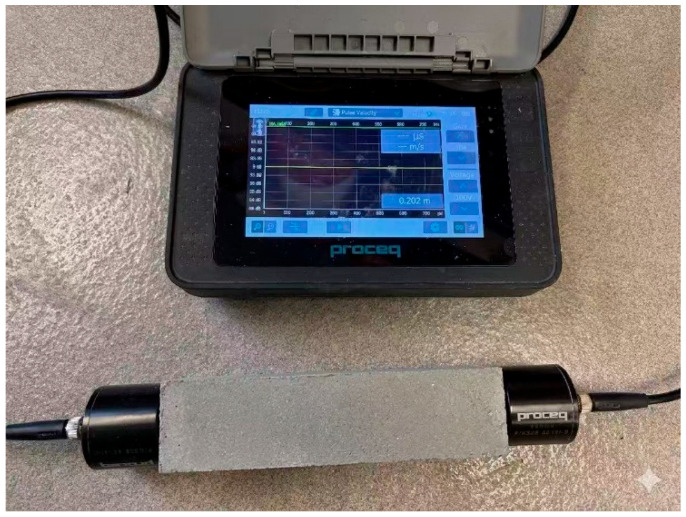
The ultrasonic pulse velocity equipment for dynamic modulus.

Dynamic modulus measurements were conducted on mortar specimens prepared with a paste volume of 27% using standard aggregate and sand conforming to GB/T 14685-2022 [61]. After casting and curing under sealed conditions, the specimens were stored at 23 ± 1 °C until testing. Figure 4 shows the ultrasonic pulse velocity equipment for dynamic modulus. The dynamic modulus was evaluated at 1, 3, 7, 14, 28, 56, and 91 days using a non-destructive ultrasonic pulse velocity (UPV) technique. Reported values represent the mean ± standard deviation of three replicate specimens.

A cradle-to-gate system boundary (A1–A3) was adopted to evaluate the embodied carbon associated with raw material extraction, processing, and transportation. Construction, service life, and end-of-life stages were excluded because they depend on application-specific assumptions that are beyond the scope of this study. The assessment was conducted using SimaPro software (v9.5) and focused on transportation-related concrete applications including bridge decks, pavements, and precast panels.

The system boundary included raw material extraction, processing, and transportation, while construction, service life, and end-of-life stages were excluded. The functional unit was defined as 1 metric ton of blended cement, and emissions were reported as kg CO_2_-equivalent per metric ton.

The A1 stage included raw material acquisition and energy production associated with OPC, fly ash, and natural pozzolans. The A2 stage considered transportation using representative haul distances and emission factors. The A3 stage included clinker production, grinding, and SCM processing operations such as crushing and drying.

**Figure 5:**
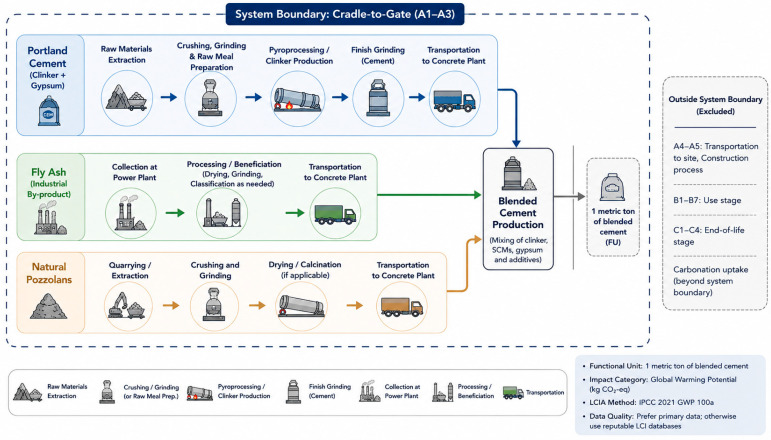
Process flow diagram of the cradle-to-gate (A1–A3) life cycle assessment model used to quantify greenhouse gas emissions of OPC and OPC + SCM. The model includes raw material extraction, material processing, transportation, and blending stages. Emissions from calcination, fuel combustion, electricity consumption, and transport are incorporated to estimate total GHG intensity and reduction relative to the OPC baseline [62].

To evaluate the effect of SCM substitution, the OPC content in baseline mixtures was partially replaced with SCMs, and the resulting changes in GHG emissions were calculated. The process flow and system boundaries used in the analysis are illustrated in Figure 5. The LCA in this study focused on transportation infrastructure applications, including bridge decks, pavements, and precast concrete panels, because the investigated natural pozzolans are locally sourced from the eastern China region and are primarily intended for regional infrastructure use.

Figure 5 illustrates the process flow and system boundary adopted in the LCA GHG reduction model. A cradle-to-gate framework (modulus A1–A3) was used to quantify greenhouse gas emissions associated with raw material extraction, constituent processing, transportation to the batching facility, and concrete mixing operations. Downstream stages, including construction, use-phase performance, carbonation uptake, and end-of-life treatment, were excluded so that the effect of binder composition on embodied carbon could be isolated more clearly. All greenhouse gas emissions are reported as kilograms of CO_2_-equivalent per cubic meter of concrete (kg CO_2_e/m^3^).

The model accounts for process emissions associated with clinker production, including limestone calcination and kiln fuel combustion, as well as electricity use for grinding and material handling, batching operations, and transportation emissions expressed on a ton–kilometer basis. Among these contributions, clinker-related emissions were found to dominate the total embodied carbon. For the reference concrete, the total embodied carbon was calculated as 382.11 kg CO_2_e/m^3^, including materials, batching, and transportation. A simple sensitivity check further showed that moderate variation in transport distance did not materially affect the comparative conclusions, because clinker production remained the dominant source of emissions.

## 3. Results and Discussion

### 3.1. Chemical and Physical Results

**Figure 6:**
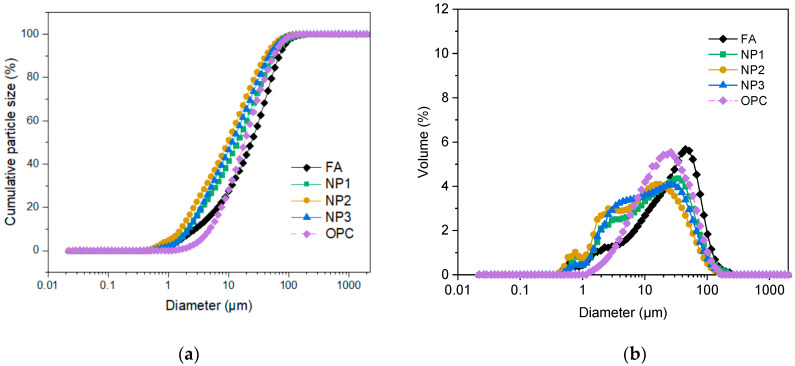
Particle size distribution curves of OPC and SCMs showing (**a**) differential volume distribution and (**b**) cumulative particle size distribution.

**Figure 7:**
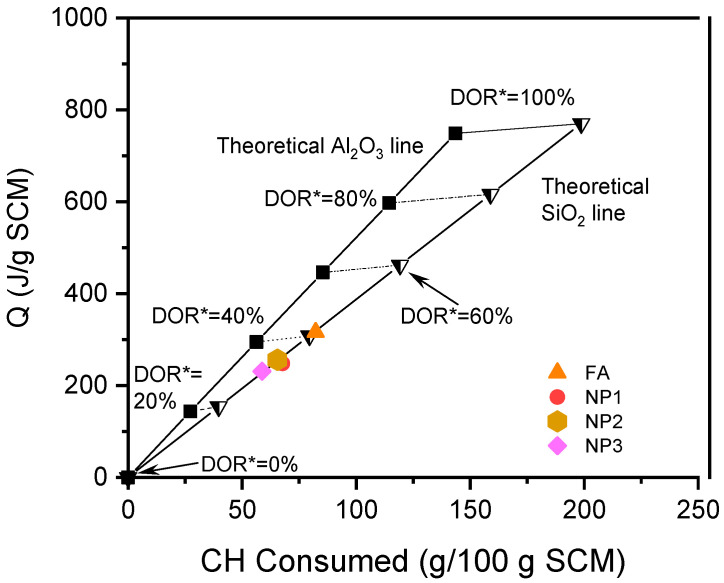
The pozzolanic reactivity results of SCMs.

The specific gravities, chemical compositions, LOI, and particle size characteristics of the tested materials are summarized in Table 3, Table 4, Table 5 and Figure 6 respectively. The pozzolanic reactivity determined using the DOR* method is presented in Figure 7. Among the investigated supplementary cementitious materials (SCMs), Class F fly ash exhibited the highest DOR* value (40.36%), whereas NP1, NP2, and NP3 showed lower reactivities of 32.81%, 28.68%, and 26.22%, respectively.

The XRF results indicate that all three natural pozzolans are silica-rich materials, with SiO_2_ contents ranging from 65% to 71%, consistent with the characteristics of natural pozzolans reported from other regions worldwide, including Turkey, Spain, and Saudi Arabia. The relatively high silica contents suggest good potential for pozzolanic reaction and durability enhancement. In contrast, the fly ash exhibited a slightly lower SiO_2_ content but higher overall pozzolanic reactivity as measured by DOR*.

Particle size analysis showed distinct differences among the SCMs (Figure 6 and Table 5). Compared with OPC, Class F fly ash exhibited a coarser particle size distribution, particularly in terms of d50 and d90. In contrast, natural pozzolans generally possessed finer median particle sizes, with NP2 exhibiting the finest overall distribution among the investigated SCMs. These differences in fineness are expected to influence particle packing, nucleation effects, water demand, and early-age hydration behavior, despite the lower intrinsic pozzolanic reactivity of the natural pozzolans. All reported values represent the average of three replicate measurements.

### 3.2. Hydration Heat

The effect of incorporating 20 vol% SCMs on hydration kinetics was evaluated through isothermal calorimetry of OPC-SCM blended systems. Among the ten candidate SCMs initially screened, three locally sourced natural pozzolans were selected for further investigation based on availability and preliminary characterization results.

**Figure 8:**
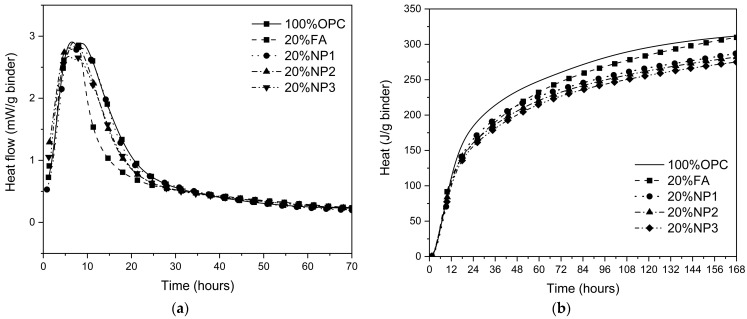
Heat evolution behavior of OPC and SCM-blended pastes showing (**a**) heat flow curves and (**b**) cumulative heat release profiles.

Figure 8 presents the heat flow and cumulative heat evolution of the tested pastes. Figure 8a illustrates the rate of heat evolution, while Figure 8b shows the accumulated heat release over the 7-day monitoring period. Heat values were normalized by binder mass to facilitate comparison among mixtures.

All tested mixtures exhibited comparable peak heat flow, with the OPC reference and FA-blended mixture reaching approximately 2.89 mW/g binder and the natural pozzolan systems averaging around 2.63 mW/g binder—a difference of approximately 0.26 mW/g binder that is not considered practically significant. Similarly, cumulative heat release after 7 days was broadly similar across all systems, with OPC and OPC + FA reaching approximately 310 J/g binder and the natural pozzolan mixtures reaching approximately 286 J/g binder, a modest reduction of approximately 8%.

The marginally lower heat release observed in the natural pozzolan systems is attributable to their reduced clinker content and moderate early-age pozzolanic reactivity, with additional contributions from differences in particle fineness, mineral composition, and amorphous phase content. Although the absolute magnitude of this reduction is modest, even a moderate decrease in heat generation rate can be practically meaningful in temperature-sensitive applications. In mass concrete elements such as bridge piers, thick foundation slabs, and dam sections, lower peak hydration temperatures reduce the risk of thermally induced cracking caused by differential temperature gradients between the core and surface. In hot-weather concreting or precast production where temperature control measures are limited, the reduced heat output of natural pozzolan systems may simplify thermal management without the need for ice additions or chilled water. Furthermore, the lower total heat release over 7 days suggests a more gradual early-age development that is compatible with applications prioritizing long-term durability over rapid strength gain, such as pavement and bridge deck construction where extended curing periods are feasible.

### 3.3. Setting Time

**Figure 9:**
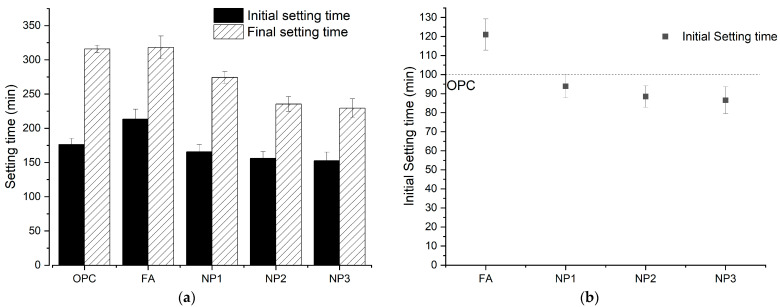
The initial and final setting results of OPC and OPC + SCM (**a**) setting time, (**b**) normalized setting time of OPC.

The setting time results for OPC and OPC + SCM systems are presented in Figure 9 and the summarized values are provided in Table 6. The results were normalized to the OPC initial setting time to facilitate comparison of hydration kinetics. OPC exhibited an initial setting time of 176.36 ± 8.76 min and a final setting time of 315.89 ± 5.68 min. Among all mixtures, FA significantly delayed the setting process, with an initial setting time of 213.45 ± 14.52 min and a final setting time of 318.17 ± 16.75 min, corresponding to approximately 121% and 101% of the OPC values, respectively. This retardation is consistent with the relatively lower early-age pozzolanic reactivity of FA, as reflected in its delayed hydration kinetics observed in the calorimetry data. In contrast, the natural pozzolans accelerated the setting process. NP1 exhibited an initial setting time of 165.55 ± 10.68 min and a final setting time of 274.33 ± 8.57 min, corresponding to approximately 94% and 87% of the OPC values, respectively. NP2 and NP3 further shortened the setting process, with initial setting times of 156.05 ± 9.87 min and 152.66 ± 12.51 min, corresponding to 89% and 87% of OPC, respectively. Their final setting times were reduced to 235.44 ± 10.74 min and 229.35 ± 13.58 min, representing reductions of approximately 25% and 27% relative to OPC. These results indicate that the incorporation of natural pozzolans promoted faster early stiffening than either OPC or FA.

The setting behavior observed in this study is consistent with previous reports showing that the influence of natural pozzolans on cement setting is highly dependent on their source characteristics. Sanjuán et al. [33] demonstrated that volcanic ash from the La Palma eruption satisfied the requirements for use as a cement constituent, although changes in setting behavior were observed with increasing replacement level. Similarly, Khan and Alhozaimy [34] reported that concretes incorporating Saudi Arabian natural pozzolans exhibited setting characteristics suitable for practical applications while maintaining satisfactory fresh properties. Together with the present results, these studies indicate that the setting response of natural pozzolans is governed by multiple factors, including mineralogical composition, particle fineness, and water absorption, rather than by replacement level alone. The relatively fine particle size and higher alkali contents of the eastern China natural pozzolans investigated in this study may have promoted early dissolution and nucleation, contributing to the accelerated setting behavior observed.

The accelerated setting behavior of the natural pozzolan systems carries practical implications for construction applications. Shorter initial setting times can be advantageous in precast concrete production, where faster form-stripping cycles improve plant throughput and reduce energy consumption associated with accelerated curing. In repair and rehabilitation applications, such as bridge deck patching or pavement joint filling, rapid setting reduces lane closure durations and minimizes traffic disruption. Additionally, in cold-weather concreting, faster setting reduces the vulnerability window during which fresh concrete is susceptible to frost damage before sufficient strength is developed. However, it should be noted that the accelerated setting observed for NP2 and NP3 may require adjustments to mixing and placement procedures, including reduced transportation time and earlier finishing operations, to ensure workability is maintained in field applications.

### 3.4. Alkaline–Silica Reaction

**Figure 10:**
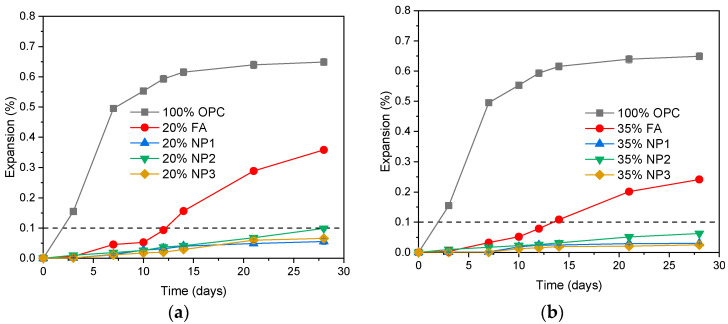
Expansion results of OPC and OPC + SCM (**a**) 20% replacement (**b**) 35% replacement.

Figure 10 presents the ASR expansion behavior of mixtures containing fly ash and natural pozzolans at SCM replacement levels of 20% and 35% by volume. The OPC reference mixture exhibited rapid expansion development, exceeding 0.50% expansion by 14 days and continuing to increase throughout the testing period, indicating severe ASR susceptibility of the reactive aggregate system. In contrast, all SCM-containing mixtures significantly reduced ASR expansion relative to the OPC control. Among the investigated SCMs, the natural pozzolan systems demonstrated substantially superior ASR mitigation performance compared with fly ash. Although FA reduced expansion compared with OPC, the FA mixtures continued to exhibit noticeable expansion growth during the testing period. At 20% replacement, the FA mixture still exceeded the commonly used 0.10% mitigation threshold at later ages, while the natural pozzolan mixtures remained substantially lower. At 35% replacement, the natural pozzolan systems maintained very low expansion values throughout the testing period, with NP1 and NP3 exhibiting the lowest overall expansions among all investigated SCMs.

**Figure 11:**
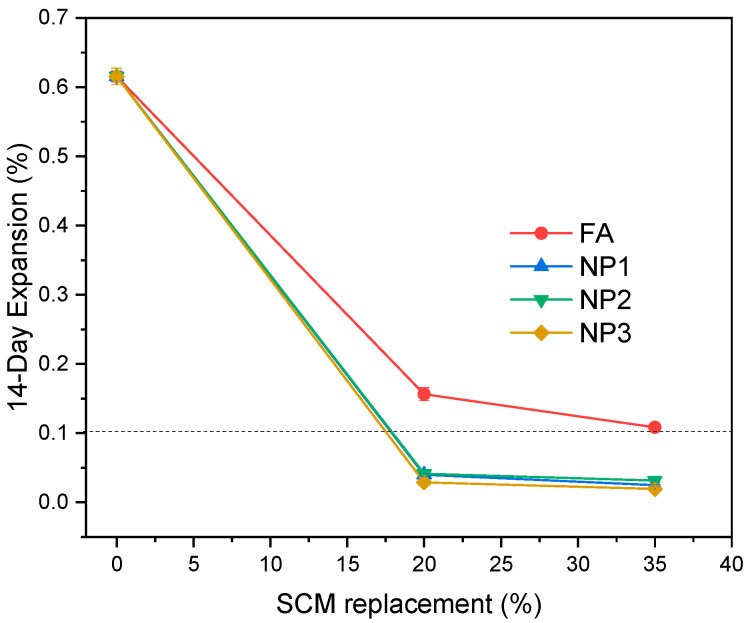
Effect of SCM replacement level on ASR expansion of FA and natural pozzolan mixtures.

To further compare the effectiveness of the SCM systems, Figure 11 and Table 7 summarize the relationship between SCM replacement level and ASR expansion. The results indicate that the natural pozzolans achieved significantly greater expansion reduction than FA at equivalent replacement levels. At both 20% and 35% replacement, NP1, NP2, and NP3 reduced expansion to values near or below the 0.10% mitigation threshold, whereas the FA systems remained noticeably higher. These results suggest that the investigated natural pozzolans provided more effective suppression of deleterious ASR expansion than conventional fly ash under the tested conditions.

**Figure 12:**
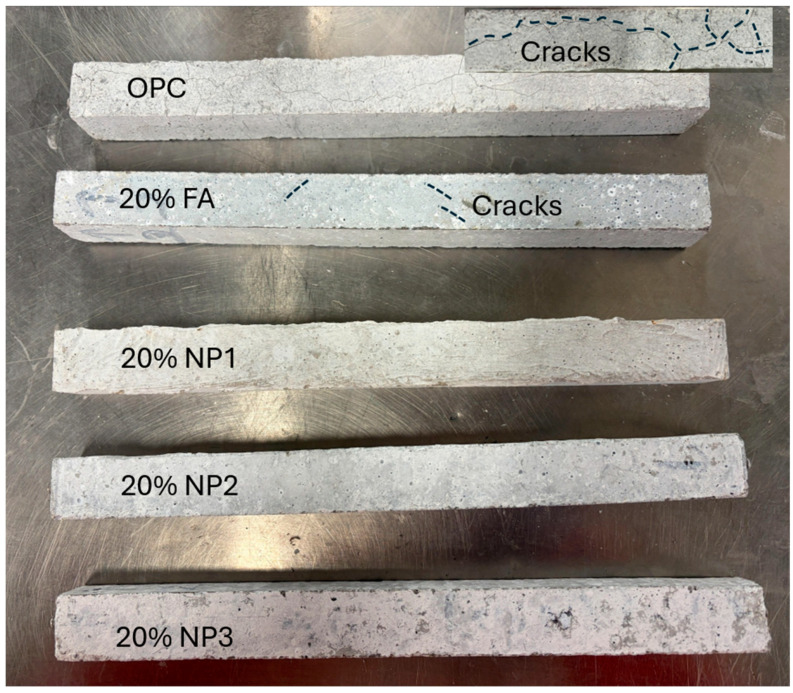
Surface cracking of OPC and SCM mixtures after accelerated ASR testing.

The improved ASR resistance of the natural pozzolan systems is likely associated with several well-established mechanisms documented in the literature. First, partial replacement of OPC with pozzolanic materials reduces the hydroxyl ion (OH^−^) concentration in the pore solution through alkali dilution, thereby limiting the driving force for deleterious silica dissolution and ASR gel formation [63]. Second, the consumption of calcium hydroxide (CH) through pozzolanic reaction lowers the alkalinity available to sustain ASR expansion, a mechanism that has been extensively documented for SCM-containing systems [64]. Third, the pozzolanic reaction products refine the pore structure and densify the interfacial transition zone, reducing the permeability to moisture and alkali ingress that sustains gel swelling [65]. In addition, the relatively high silica contents (65–71%) and fine particle size distributions of the investigated natural pozzolans may have promoted more complete pozzolanic reaction and more thorough modification of pore solution chemistry compared with natural pozzolans from other geological sources reported in the literature. Differences in mitigation effectiveness among NP1, NP2, and NP3 are likely related to variations in amorphous silica content, fineness, and overall pozzolanic reactivity, as evidenced by the DOR* results presented in Section 3.1. It should be noted that these mechanisms were not directly confirmed through pore solution analysis or microstructural characterization in the present study, and future work incorporating such techniques would provide more direct evidence for the proposed explanations. The visual cracking observations shown in Figure 12 further support the expansion measurements. Severe surface cracking was observed in the OPC specimens, while visible cracking was still present in the FA mixtures. In contrast, the natural pozzolan mixtures exhibited substantially reduced visible cracking and improved surface integrity after accelerated ASR exposure, indicating enhanced resistance to ASR-related deterioration. Overall, the ASR results demonstrate that the investigated natural pozzolans, particularly NP1 and NP3, can provide superior ASR mitigation performance compared with conventional fly ash.

The superior ASR mitigation observed for the Nanjing natural pozzolans in this study can be interpreted within the broader context of natural pozzolan durability research. While ASR-specific data are not reported in Çavdar and Yetgin [32], Khan and Alhozaimy [34], or Sanjuán et al. [33], these studies consistently demonstrate that natural pozzolans improve transport-related durability metrics through pore structure refinement and pozzolanic consumption of calcium hydroxide. Khan and Alhozaimy [34] reported that chloride permeability of concrete containing 15–25% Saudi natural pozzolan decreased substantially at ages beyond 90 days, reaching values well below those of plain concrete, and XRD analysis confirmed the progressive conversion of crystalline calcium hydroxide to amorphous C–S–H. Sanjuán et al. [33] similarly reported that incorporation of La Palma volcanic ash reduced capillary sorptivity and increased electrical resistivity at later ages, with mortars containing 40% ash exhibiting capillary absorption coefficients approximately 41% lower than the OPC reference. These pore refinement and calcium hydroxide consumption mechanisms are fundamentally the same mechanisms proposed to underlie ASR mitigation in the present study, namely, reduction in pore solution alkalinity, alkali dilution, and densification of the interfacial transition zone. The relatively high SiO_2_ contents of the natural pozzolans (65–71%) from eastern China compared to the Saudi (41–43%) and La Palma (45%) materials may account for their particularly effective ASR suppression, as greater reactive silica availability promotes more complete pozzolanic reaction and more thorough modification of pore solution chemistry.

### 3.5. Compressive Strength Development

**Figure 13:**
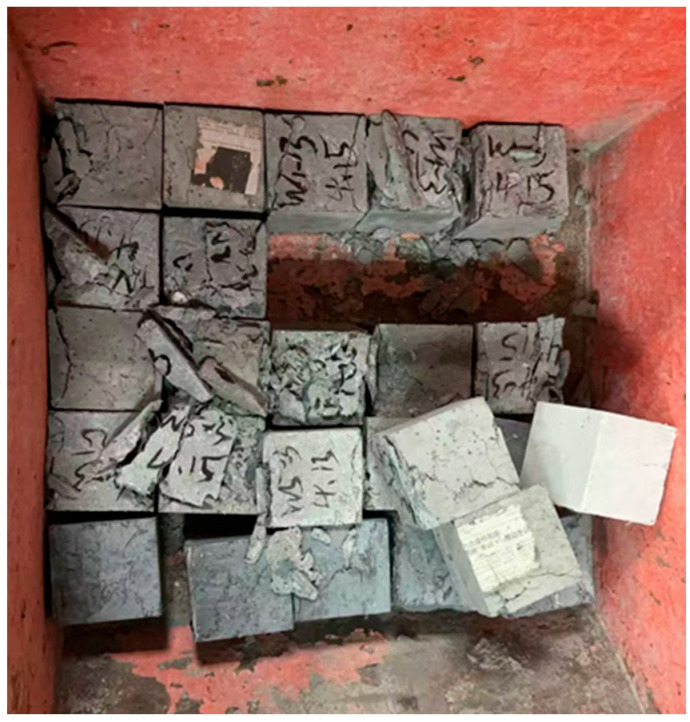
The fracture patterns of tested samples.

**Figure 14:**
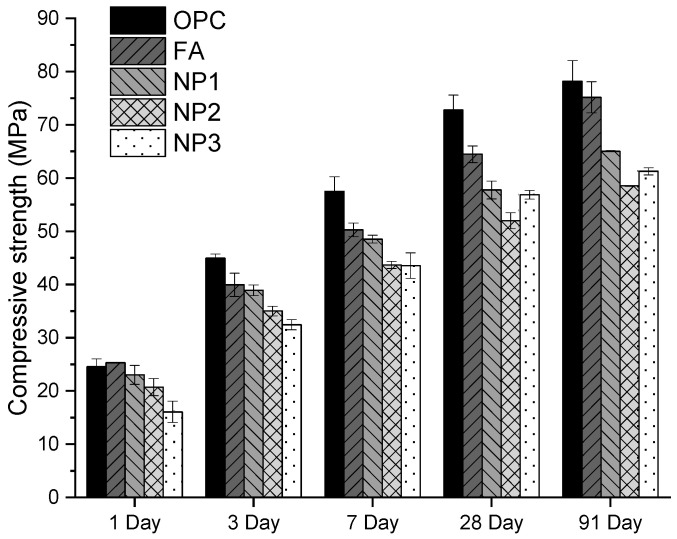
Comparison of the compressive strength of OPC and OPC + SCM.

Figure 13 presents the representative fracture patterns of the tested samples. The compressive strength development of OPC and OPC + SCM mixtures at 1 to 91 days are shown in Figure 14 and Table 8. Three distinct behavioral trends emerge from the results. First, all SCM-containing mixtures exhibited lower early-age strength than OPC, reflecting the dilution of clinker content and the slower activation of pozzolanic reactions at early ages. FA and NP1 showed the smallest early-age penalties, maintaining approximately 87–103% of OPC strength at 1–7 days, while NP2 and NP3 exhibited more pronounced early-age reductions, reaching only 65–84% of OPC at 1 day. Second, all mixtures demonstrated continuous strength gain with curing age, with the strength gap relative to OPC narrowing progressively from 7 days onward. This trend reflects the gradual activation of pozzolanic reactions and the associated densification of the cementitious matrix through secondary C-S-H formation. By 91 days, FA approached the OPC control at 96%, while NP1, NP2, and NP3 reached 83%, 75%, and 78% of OPC, respectively. Third, NP1 consistently outperformed NP2 and NP3 across all ages, which is consistent with its higher pozzolanic reactivity indicated by the DOR* results in Section 3.1.

From a strength activity index perspective [66,67] in Table 9, NP1 and NP3 met the 75% criterion by 28 and 91 days respectively, while NP2 reached 74.9% at 91 days, marginally below the threshold. The moderate but steady mechanical development of the natural pozzolan systems indicates meaningful long-term pozzolanic contribution, and their performance is considered acceptable for applications where long-term durability and carbon reduction are prioritized over early-age strength, such as pavement and bridge deck construction. Taken together with the superior ASR mitigation performance demonstrated in Section 3.4, these results indicate that NP1 and NP3 can serve as effective fly ash alternatives combining durability performance with acceptable long-term mechanical development.

The mechanical performance trends observed in the present study are consistent with patterns reported for natural pozzolans from other regions. Khan and Alhozaimy [34] found that concrete containing 15% Saudi natural pozzolan exhibited compressive strength at 7 and 28 days corresponding to approximately 77% and 78% of the plain concrete reference, respectively, meeting the ASTM C618 Class N strength activity index requirement of 75%. In the present study, NP1 and NP3 achieved SAI values exceeding 75% by 28 and 91 days, respectively, which is broadly comparable to the performance reported by Khan and Alhozaimy despite the different replacement levels and testing methodologies. Similarly, Sanjuán et al. [33] reported that blended cements containing 25% La Palma volcanic ash achieved a 28-day SAI of 84.3%, meeting the EN 450-1 requirement; their results also confirmed that compressive strength decreased progressively with increasing ash replacement level, a trend consistent with the behavior of NP2 and NP3 in the present study at higher replacement levels. The natural pozzolans from northeastern Turkey investigated by Çavdar and Yetgin [32] demonstrated 7-day mortar compressive strengths of 6.7–11.0 MPa in a lime-pozzolan system, with pozzolanic activity correlating positively with SiO_2_ content—a finding that aligns with the stronger mechanical performance of NP1 (SiO_2_ = 66.4%) relative to NP2 (SiO_2_ = 71.2%, but lower Al_2_O_3_) and NP3 in the present study. Across these globally diverse datasets, a consistent picture emerges: natural pozzolans generally exhibit lower early-age strength than OPC or fly ash, with gradual strength recovery at later ages driven by continued pozzolanic reaction, and higher SiO_2_ content tends to be associated with stronger mechanical development.

### 3.6. Dynamic Modulus Development

**Figure 15:**
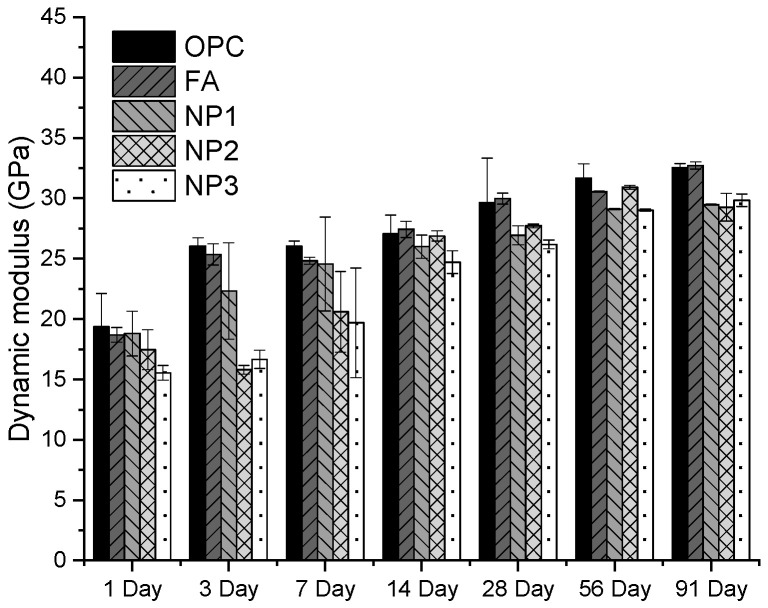
Comparison of the dynamic modulus of OPC and OPC + SCM.

The dynamic modulus development of OPC and OPC + SCM mortar mixtures from 1 to 91 days is shown in Figure 15 and Table 10. Two principal trends characterize the dynamic modulus results. First, the SCM systems separated into two distinct groups at early ages: FA and NP1 maintained stiffness development closely tracking OPC (97% and 97% of OPC at 1 day, respectively), whereas NP2 and NP3 exhibited substantially lower early-age stiffness, reaching only 90% and 80% of OPC at 1 day and dropping further to 61% and 64% at 3 days. This grouping mirrors the early-age compressive strength trends observed in Section 3.4 and reflects the same underlying cause—the more sluggish early pozzolanic activation of NP2 and NP3 relative to NP1 and FA, as indicated by their lower DOR* values in Section 3.1. Second, the differences among all mixtures converged substantially with continued curing. By 28 days, NP2 had recovered to 94% of OPC dynamic modulus, comparable to NP1 (91%) and NP3 (88%), indicating that the delayed pozzolanic reaction of these systems progressively contributed to matrix stiffening. At 91 days, all three natural pozzolans reached 90–92% of OPC, while FA slightly exceeded the OPC reference at 101%, consistent with its more complete long-term pozzolanic development.

The convergence of dynamic modulus values at later ages, despite the pronounced early-age differences, suggests that the natural pozzolan systems develop a comparable elastic framework to OPC over time. This is particularly relevant for structural applications where stiffness under service loading is the governing criterion rather than early-age strength, and where sufficient curing time is available before load application.

### 3.7. Life Cycle Analysis

**Figure 16:**
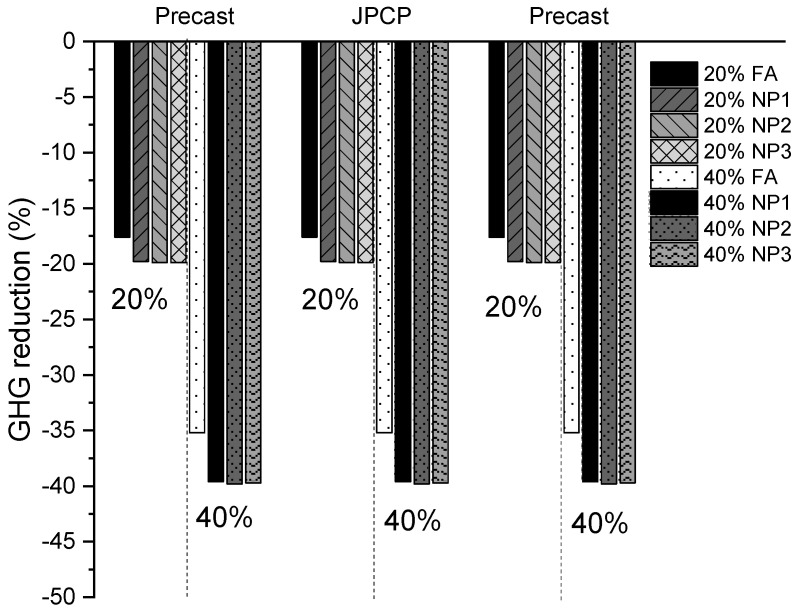
Greenhouse gas reductions compared to 20 and 40% interground SCM replacements.

The embodied carbon results for the SCM-blended systems are summarized in Figure 16. At the 20% replacement level, the embodied carbon decreased from 382.11 kg CO_2_e/m^3^ for the OPC reference mixture to 306.62 kg CO_2_e/m^3^ for the fly ash system and 306.84 kg CO_2_e/m^3^ for the natural pozzolan system, corresponding to reductions of approximately 19.8% and 19.7%, respectively. At the 40% replacement level, the embodied carbon was further reduced to 231.12 kg CO_2_e/m^3^ for fly ash and 231.58 kg CO_2_e/m^3^ for natural pozzolan, corresponding to reductions of approximately 39.5% and 39.4%, respectively. These results indicate that increasing SCM replacement from 20% to 40% substantially enhances the reduction in embodied carbon.

Two main trends emerge from these results. First, embodied carbon reduction is governed primarily by the decrease in clinker fraction, with higher replacement levels producing markedly larger reductions in total emissions. Second, the differences between fly ash and natural pozzolan are very small under the present assumptions, indicating that SCM type has a secondary influence compared with total clinker replacement. Although transportation emissions were somewhat higher for fly ash because of the longer assumed transport distance, their overall contribution remained negligible relative to clinker-related emissions. Overall, these findings confirm that clinker factor reduction is the dominant driver of embodied carbon mitigation, and that both fly ash and natural pozzolans can provide substantial greenhouse gas reduction benefits when used as partial cement replacements.

## 4. Conclusions

This study systematically evaluated three locally sourced natural pozzolans from eastern China as potential alternatives to Class F fly ash, with emphasis on alkali–silica reaction (ASR) mitigation, hydration behavior, mechanical performance, setting characteristics, and embodied carbon reduction. Based on the experimental results, the following conclusions can be drawn:

The investigated natural pozzolans exhibited excellent potential as supplementary cementitious materials. All three natural pozzolans were silica-rich (65–71%) and possessed relatively fine particle size distributions, although their overall pozzolanic reactivity (DOR*) was lower than that of Class F fly ash.

Natural pozzolans provided substantially superior ASR mitigation compared with Class F fly ash. At equivalent volumetric replacement levels, NP1 and NP3 maintained expansion values near or below the commonly adopted 0.10% threshold and exhibited significantly reduced surface cracking. Despite their lower DOR* values, the natural pozzolans consistently outperformed fly ash in suppressing ASR expansion, indicating that ASR mitigation is governed not only by overall pozzolanic reactivity but also by chemical composition, particle characteristics, and pore solution modification.

The improved durability was achieved while maintaining acceptable long-term mechanical performance. Although the incorporation of natural pozzolans reduced early-age hydration heat, compressive strength, and dynamic modulus compared with OPC and fly ash, continuous strength and stiffness development was observed throughout curing. At 91 days, compressive strength and dynamic modulus reached up to 83% and 92% of OPC, respectively, demonstrating satisfactory long-term mechanical performance.

Natural pozzolans accelerated setting while reducing embodied carbon emissions. Unlike Class F fly ash, which delayed setting, all three natural pozzolans shortened both initial and final setting times. In addition, the cradle-to-gate life cycle assessment demonstrated that replacing clinker with locally sourced natural pozzolans substantially reduced embodied carbon emissions, providing environmental benefits comparable to those achieved with fly ash.

Overall, the results demonstrate that locally sourced natural pozzolans, particularly NP1 and NP3, are promising regional alternatives to Class F fly ash for durable and low-carbon cement-based systems. More importantly, this study demonstrates that superior ASR mitigation can be achieved despite lower measured pozzolanic reactivity, highlighting that durability performance should be evaluated using multiple criteria rather than relying solely on DOR*. The unified evaluation framework developed in this study, integrating hydration, setting, mechanical performance, ASR mitigation, and embodied carbon assessment, provides a transferable methodology for assessing locally available supplementary cementitious materials in regions where fly ash availability is declining.

## Figures and Tables

**Table 1:** The mixture proportion (kg/m^3^).

No.	Cement	SCM	Water
OPC	1302.97	0.00	586.34
FA	1042.38	174.60	547.54
NP1	1042.38	195.20	557.69
NP2	1042.38	182.79	551.98
NP3	1042.38	189.47	554.98

**Table 2:** Mortar mixture proportions (kg/m^3^ mortar).

Mix	Cement	SCM	Water	Sand
OPC	651.49	0.00	293.17	1325.00
FA	521.19	87.30	273.77	1325.00
NP1	521.19	97.60	278.85	1325.00
NP2	521.19	91.40	275.99	1325.00
NP3	521.19	94.74	277.49	1325.00

**Table 3:** Specific gravity values of tested materials (g/cm^3^).

SCM	Specific Gravity
FA	2.11 ± 0.07
OPC	3.15 ± 0.00
NP1	2.36 ± 0.01
NP2	2.21 ± 0.09
NP3	2.29 ± 0.05

**Table 4:** Chemical composition of cementitious materials.

%	OPC	FA	NP1	NP2	NP3
SiO_2_	20.55	60.36	66.40	71.24	65.18
Al_2_O_3_	4.31	21.52	15.00	12.72	18.89
Fe_2_O_3_	2.28	5.04	1.19	1.47	1.49
CaO	60.89	5.40	5.05	2.65	5.59
MgO	1.62	1.43	0.23	0.12	1.47
SO_3_	2.55	0.06	0.10	0.19	0.08
Na_2_O	0.21	1.48	3.31	2.93	1.61
K_2_O	0.48	0.85	4.48	5.12	1.56
TiO_2_	0.19	1.16	0.04	0.01	0.04
P_2_O_5_	0.1	0.25	0.00	0.09	0.05
ZnO	0.01	0.06	0.01	0.00	0.01
Mn_2_O_3_	0.07	0.02	0.01	0.00	0.07
Cl	0.00	0.01	0.00	0.02	0.00
LOI	2.71	2.41	4.17	3.45	3.96

**Table 5:** Characteristic particle diameters (d10, d50, and d90) of the investigated materials.

Cementitious Name	d10 (µm)	d50 (µm)	d90 (µm)
OPC	5.12 ± 0.37	18.10 ± 0.47	55.68 ± 0.12
FA	2.98 ± 1.23	23.01 ± 1.38	68.62 ± 1.1
NP1	2.27 ± 0.64	13.63 ± 0.40	54.36 ± 0.02
NP2	1.61 ± 0.75	10.09 ± 0.35	46.60 ± 0.14
NP3	2.27 ± 0.93	11.57 ± 0.44	48.20 ± 0.47

**Table 6:** Setting time of OPC and OPC + SCM.

	OPC	FA	NP1	NP2	NP3
Initial setting time/min	176.36 ± 8.76	213.45 ± 14.52	165.55 ± 10.68	156.05 ± 9.87	152.66 ± 12.51
Final setting time/min	315.89 ± 5.68	318.17 ± 16.75	274.33 ± 8.57	235.44 ± 10.74	229.35 ± 13.58

**Table 7:** 14 Day expansion of alkaline–silica reaction.

Replacement	OPC	FA	NP1	NP2	NP3
20%	0.62 ± 0.01	0.16 ± 0.01	0.04 ± 0.00	0.04 ± 0.00	0.03 ± 0.00
35%	0.62 ± 0.01	0.11 ± 0.00	0.02 ± 0.00	0.03 ± 0.00	0.02 ± 0.00

**Table 8:** Compressive strength of OPC and SCM-blended systems at different ages (MPa, mean ± SD).

Mixture	1 Day	3 Days	7 Days	28 Days	91 Days
OPC	24.57 ± 1.47	44.96 ± 0.82	57.46 ± 2.99	72.78 ± 2.81	78.19 ± 3.98
FA	25.30 ± 0.10	39.93 ± 2.20	50.26 ± 1.39	64.46 ± 1.56	75.17 ± 2.93
NP1	23.01 ± 1.78	38.90 ± 1.06	48.51 ± 0.79	57.75 ± 1.67	65.03 ± 0.09
NP2	20.71 ± 1.61	35.01 ± 0.91	43.66 ± 0.72	51.98 ± 1.50	58.53 ± 0.56
NP3	16.06 ± 2.00	32.43 ± 0.98	43.55 ± 2.58	56.85 ± 0.82	61.26 ± 1.02

**Table 9:** Strength Activity Index (SAI) of fly ash and natural pozzolan blended systems at 28 and 91 days, relative to OPC, and classification based on the 75% criterion [66].

Mixture	28-Day SAI (%)	28-Day Classification	91-Day SAI (%)	91-Day Classification
FA	88.6	Meets criterion	96.1	Meets criterion
NP1	79.4	Meets criterion	83.2	Meets criterion
NP2	71.4	Below criterion	74.9	Near criterion
NP3	75.1	Meets criterion	78.3	Meets criterion

**Table 10:** Dynamic modulus of OPC and SCM-blended systems at different ages (GPa, mean ± SD).

Mixture	1 Day	3 Days	7 Days	28 Days	91 Days
OPC	19.35 ± 2.77	26.04 ± 0.70	26.02 ± 0.44	29.64 ± 3.69	32.56 ± 0.32
FA	18.69 ± 0.62	25.35 ± 0.88	24.83 ± 0.29	29.98 ± 0.46	32.72 ± 0.29
NP1	18.79 ± 1.85	22.32 ± 3.99	24.56 ± 3.90	26.93 ± 0.80	29.47 ± 0.06
NP2	17.46 ± 1.65	15.79 ± 0.35	20.60 ± 3.34	27.72 ± 0.15	29.26 ± 1.15
NP3	15.54 ± 0.61	16.65 ± 0.75	19.70 ± 4.54	26.18 ± 0.37	29.83 ± 0.52

## Data Availability

The original contributions presented in this study are included in the article. Further inquiries can be directed to the corresponding authors.
